# The impact of preventive measures on the burden of femoral fractures – a modelling approach to estimating the impact of fall prevention exercises and oral bisphosphonate treatment for the years 2014 and 2025

**DOI:** 10.1186/s12877-016-0247-9

**Published:** 2016-04-01

**Authors:** Petra Benzinger, Clemens Becker, Chris Todd, Florian Bleibler, Dietrich Rothenbacher, Hans-Helmut König, Kilian Rapp

**Affiliations:** Department of Clinical Gerontology, Robert Bosch Krankenhaus, Auerbachstrasse 110, 70376 Stuttgart, Germany; School of Nursing, Midwifery & Social Work and Manchester Academic Health Science Centre, The University of Manchester, Oxford Road, Manchester, M13 9PL UK; Department of Health Economics and Health Services Research, Hamburg Centre for Health Economics, University Medical Center Hamburg-Eppendorf, 20246 Hamburg, Germany; Institute of Epidemiology and Medical Biometry, Ulm University, Helmholzstrasse 22, 89081 Ulm, Germany

**Keywords:** Femoral fractures, Prevention, Epidemiology, Osteoporosis, Falls

## Abstract

**Background:**

Due to the demographic transition with a growing number of old and oldest-old persons the absolute number of fragility fractures is expected to increase in industrialized countries unless effective preventive efforts are intensified. The main causes leading to fractures are osteoporosis and falls. The aim of this study is to develop population based models of the potential impact of fall-prevention exercise and oral bisphosphonates over the coming decade.

**Methods:**

The German federal state of Bavaria served as the model population.

Model interventions were limited to community-dwelling persons aged 65 years and older. Models are based on fall-prevention exercise being offered to all persons aged 70 to 89 years and oral bisphosphonate treatment offered to all persons with osteoporosis as defined by a T-score of ≤ − 2.5. Treatment effect sizes are estimated from meta-analyses. Reduction in all femoral fractures in the population of community-dwelling persons aged 65 years and older is the outcome of interest. A spreadsheet-based modelling approach was used for prediction.

**Results:**

In 2014, reduction of femoral fractures by 10 % required 21 % of all community-dwelling persons aged 70–89 to participate in fall-prevention exercise, or 37 % of those with osteoporosis to receive oral bisphosphonates. Without intervention, demographic changes will result in a 24 % increase in femoral fractures by 2025. To lower the increase of fractures between 2014 and 2025 to 10 %, fall-prevention-exercise participation rate needs to be 25 % and bisphosphonate treatment rates 41 %, whereas to hold the 2025 rates flat at 2014 rates require 43 % fall-prevention-exercises participation, and is not achievable using oral bisphosphonates.

**Conclusions:**

Unrealistic high treatment and participation rates of the two analysed measures are needed to achieve substantial effects on the expected burden of femoral fractures at present and in the future.

## Background

With increasing age, the risk of fragility fractures such as femoral or vertebral fractures increases exponentially. Femoral fractures are the most common, costly, and resource consuming type of fragility fractures [1]. The secular trend in rates of femoral fractures remains unclear. While some countries report a lowering of femoral fracture rates over the last decade, other countries such as Germany report unchanged rates [2, 3]. Due to the growing number of old and very old persons in industrialised countries, the absolute number of fractures is expected to rise substantially over the coming decades; a trend that could only be reversed if prevention receives more attention [1, 4]. Therefore, it is of interest to explore what impact wider implementation of existing preventive measures could have on (i) the situation today and (ii) the projected increase in fractures over the coming years.

The two main underlying mechanisms of fragility fracture are osteoporosis and falls [5]. For both, there are evidence-based preventive interventions available. Several pharmaceutical agents offer effective treatment options to improve low bone mineral density (BMD) in osteoporosis. Best evidence exists for bisphosphonates, which are regarded as first line medication [6, 7]. Clearly, this treatment addresses only persons with decreased BMD. For the prevention of falls, various strategies have been shown to be effective [8]. A variety of exercise-based programmes have been developed for the general older population, e.g. persons across the spectrum of risk of falls. Most of these programmes are based on balance and strength training, delivered either in groups or individually [9].

The aim of this paper was to estimate the potential impact of these two interventions, (a) treating osteoporosis with oral bisphosphonates, and (b) preventing falls by Fall Prevention Exercise (FPE), on the expected burden of femoral fractures in community-dwelling persons 65 years and older at present and in 2025. We excluded nursing home residents as their fracture risk differs from that of community-dwelling persons [10].

## Methods

The population structure of Bavaria, Germany was used for our modelling approach to provide a model for central Europe. This population was chosen because of the availability of age- and gender-stratified fracture rates [10], and official population data, both current and projected to 2025. Bavaria is one federal state of Germany with around 12.7 million inhabitants, with an estimated 2.42 million persons aged 65 years or older at the end of 2014 [11]. By 2025, this number is expected to increase to 2.83 million persons. We were interested in the impact of two different prevention strategies to lower the number of femoral fractures in 2014 and to lower the expected increase of femoral fractures between 2014 and 2025: fall prevention by exercise and osteoporosis treatment by bisphosphonate medication. The analyses were based on the following assumptions (see Appendix 1):

### Fracture rates and demographic development

Age- and gender-specific femoral fracture rates of community-dwelling people aged 65 years and older were derived from a publication which used health claims data from the *Allgemeine Ortskrankenkasse Bayern* (AOK Bavaria) between 01.01.2004 and 30.06.2009. The AOK is a statutory health and long-term care insurance company and covers nearly 50 % of the Bavarian population aged 65 years and over. Health insurance and long-term care insurance, are compulsory in Germany. These data permitted us to exclude those in residential care, so as to estimate rates for independent community-dwelling older persons only [10]. Six age-categories were used for men and women each (65–69, 70–74, 75–79, 80–84, 85–89, and 90 years and older; see Appendix 2). The same age- and gender-specific fracture rates were used for calculations for the years 2014 and 2025. The population estimates for 2025 were provided on request by the federal statistics office of Bavaria.

### Fall-prevention exercise (FPE)

Estimates for the reduction of fractures by FPE are based on a meta-analysis of fall prevention strategies in community-dwelling older people (Rate Ratios 0.39, 95 % Confidence Interval (CI) 0.23 to 0.66) [12]. FPE is assumed to target only persons aged 70 to 89 years. The mean age in FPE studies is about 77 years. Real-world observation shows that persons younger than 70 years show little interest in FPE while those older than 89 years often are too frail to participate. To assume a realistic scenario, the intervention was focused on those interested and able to participate.

### Bisphosphonate treatment

Estimates for the reduction of femoral fractures attributable to oral bisphosphonate treatment are based on a meta-analysis (Relative Risk 0.58, 95 % CI 0.42, 0.80) [13]. The effect size of bisphosphonates in men is kept identical to that in women. Data about the efficacy of bisphosphonates in men are rare but support such an assumption [14–17]. Bisphosphonate treatment is limited to people with osteoporosis based on BMD values (T-score ≤ −2.5). Concomitant risk factors modifying the threshold for treatment were not considered.

#### Analyses

In a first step we estimated the number of persons that need to receive the intervention of interest (respectively, fall prevention by exercise or osteoporosis treatment) in order to reduce the number of femoral fractures in 2014 by 10, 15, 20, and 25 % in the community-dwelling population aged 65 years and older. In a second step, the number of persons was estimated that would need to receive each intervention of interest in order to keep the number of femoral fractures stable in 2025 compared to 2014 (0 % increase) or to limit the expected increase to 15 %, 10 %, 5 % or 0 %.

##### Community-dwelling persons (N_all_)

In order to exclude the nursing home population in our projections, we used age-gender-stratified Bavarian institutionalisation rates in order to calculate the estimated number of community-dwelling persons (N_all_) in 2014 and 2025, assuming that institutionalisation rates remain stable over time.

##### Number (FF_all_) and rates (FR_all_) of femoral fractures

The total number of femoral fractures (FF_all_) was calculated for the years 2014 and 2025 based on age- and gender-specific fracture rates and the corresponding numbers of persons, corrected as described above for nursing home residents. The following analyses on the reduction of the total number of femoral fractures (*FF*_*all*_) are based on these estimates. Overall femoral fracture rates were calculated by dividing the total number of femoral fractures by the total number of community-dwelling persons (*FF*_*all*_/N_all_).

##### Number of femoral fractures to be prevented (PFF)

The number of femoral fractures to be prevented (PFF) was calculated

- for the years 2014 (PFF (2014)) as follows:

x% reduction: PFF (2014) = *FF*_*all*_ * x/100

Calculations were performed for x = 10, 15, 20, and 25 % reduction of FF.

- for the year 2025 (PFF (2025)) as follows:

increase of fractures between 2014 and 2025 by y%: PFF (2025) = *FF*_*all*_ (2025) – (*FF*_*all*_ (2014) * (1.0 + y/100))

Calculations were performed for y = 0, 5, 10, and 15 % increase of FF between 2014 and 2025.

##### Persons with osteoporosis (N_osteoporosis_)

Osteoporosis was defined as a BMD T-score ≤ −2.5 [18, 19]. Age- and gender-specific prevalence of osteopenia and osteoporosis was used from the Rotterdam study to estimate the number of community-dwelling persons 65 years or older with normal BMD, with osteopenia and with osteoporosis in Bavaria on 31st December 2014 and on 31st December 2025 [20]. Five age-categories were used for men and women each (65–69, 70–74, 75–79, 80–84, 85 years and older; see Appendix 3). The calculated N_osteoporosis_ was 561,439 for 2014, and 666,902 for 2025 (Table 1).

**Table 1:** Estimated number of community-dwelling older persons in Bavaria and estimated number of fractures

Year	2014	2025	Increase^d^
Persons aged 65 years and older (*N*)	2,416,531	2,830,756	17.1 %
Persons aged 70 – 89 years (*N*/%^a^)	1,723,729 (71.3)	1,881,387 (66.5)	9.1 %
Persons with osteoporosis^c^ aged 65 years and older (*N*/%^a^)	561,439 (23.2)	666,902 (23.6)	18.8 %
Femoral fractures in persons aged 65 years and older (*N*)	17,119	21,263	24.2 %
Femoral fractures in persons aged 70–89 years (*N*/%^b^)	13,439 (78.5)	15,917 (74.9)	18.4 %
Femoral fractures in persons with osteoporosis^c^ aged 65 years and older (*N*/%^b^)	11,055 (64.6)	13,938 (65.6)	26.1 %

^a^ refers to % among all persons 65 years and older

^b^ refers to % among all femoral fractures in persons aged 65 years and older

^c^ (% among all persons with osteoporosis as defined by T-score ≤ −2.5)

^d^ increase between 2014 and 2025

##### Number (FF_osteoporosis_) and rates (FR_osteoporosis_) of femoral fractures in persons with osteoporosis

We used relative risks for a femoral fracture of 4.36 for osteopenia and 15.57 for osteoporosis compared to persons with normal BMD [21]. Age- and gender-specific rates were calculated by dividing FF_osteoporosis_ by N_osteoporosis_.

Results of described calculations are displayed in Table 1.

##### Percentage of the population aged 70–89 years required to reduce the absolute number of femoral fractures by fall-prevention exercise (FPE)

The number of persons aged 70 to 89 years (N_70–89_) was estimated for N_all_. The number of femoral fractures in this population (FF_70–89_) and their overall fracture rate (FR_70–89_) was calculated as described above. The number of femoral fractures prevented (PFF) remained unchanged as calculated above.

Percentage of persons participating in FPE in relation to all persons aged 70 to 89 years of age = PFF/((1-RR) * FR_70–89_)/N_70–89_).

*The percentage of persons with osteoporosis who have to be treated with bisphosphonates in order to reduce the absolute number of femoral fractures was calculated the following way:*

Percentage of persons treated with bisphosphonates in relation to all persons with osteoporosis = PFF/((1-RR) * *FR*_osteoporosis_)/N_osteoporosis_).

Results of described calculations are displayed in Tables 2 and 3.

**Table 2:** Percentage of persons required to undertake one of the two interventions in order to lower the number of femoral fractures (prevented fraction) in community-dwelling persons aged 65 years and older in 2014

Fall prevention exercise (FPE)	Percentage of persons aged 70 to 89 years needed to participate in order to achieve the targeted reduction			
	Assumed reduction in 2014 by			
	10 %	15 %	20 %	25 %
Prevented fraction based on a relative risk of 0.39	20.9	31.3	41.8	52.2
*Sensitivity analysis*				
*Lower boundary of 95 % CI: 0.22*	*16.3*	*24.5*	*32.7*	*40.8*
*Upper boundary of 95 % CI: 0.66*	*37.5*	*56.2*	*74.9*	*93.7*
Bisphosphonates	Percentage of persons aged 65 years and older with osteoporosis^b^ needed to receive medication in order to achieve the targeted reduction			
	Reduction in 2014 by			
	10 %	15 %	20 %	25 %
Prevented fraction based on a relative risk of 0.58	36.9	55.3	73.7	92.2
*Sensitivity analysis*				
*Lower boundary of 95 % CI: 0.42*	*26.7*	*40.1*	*53.4*	*66.7*
*Upper boundary of 95 % CI: 0.80*	*77.4*	^a^	^a^	^a^

^a^ > 100 %; ^b^ (T-score ≤ −2.5)

**Table 3:** Percentage of persons required to undertake one of the two interventions in 2025 in order to lower the expected increase in the absolute number of femoral fractures^a^ in community-dwelling persons aged 65 years and older to 15/10/5/0 % compared to 2014

Fall prevention exercise	Percentage of persons aged 70 to 89 years needed to participate in order to achieve the targeted reduction			
	Reduction of expected increase between 2014 and 2025 to			
	15 %	10 %	5 %	0 %
Prevented fraction based on a relative risk of 0.39	16.2	25.1	33.9	42.7
*Sensitivity analysis*				
*Lower boundary of 95 % CI: 0.22*	*12.7*	*19.6*	*26.5*	*33.4*
*Upper boundary of 95 % CI: 0.66*	*29.1*	*44.9*	*60.8*	*76.6*
Bisphosphonates	Percentage of persons aged 65 years and older with osteoporosis^c^ needed to receive medication in order to achieve the targeted reduction			
	Reduction of expected increase between 2014 and 2025 to			
	15 %	10 %	5 %	0 %
Prevented fraction based on a relative risk of 0.58	26.9	41.5	56.2	70.8
*Sensitivity analysis*				
*Lower boundary of 95 % CI: 0.42*	*19.5*	*30.1*	*40.7*	*51.3*
*Upper boundary of 95 % CI: 0.80*	*56.7*	*87.3*	^b^	^b^

^a^ expected increase: 24.2 %; ^b^ > 100 %; ^c^ (T-score ≤ −2.5)

Sensitivity analyses were performed using the upper and lower limits of the 95 % confidence intervals of the effect sizes for fall-prevention exercise and bisphosphonate treatment.

##### Number of persons and number of groups required to perform fall prevention exercise in Munich

The share of the Munich population on the total Bavarian population 65 years and older was calculated based on data provided for December 31th 2013. Fall-prevention exercise groups were assumed to consist of 15 persons each [22].

Calculations were performed using a spreadsheet based modelling approach implemented in Microsoft Excel.

## Results

The modelling approach was employed in the German federal state of Bavaria. In 2014, about 2.42 million persons aged 65 years and older are estimated to live in Bavaria, of whom 4.5 % live in nursing homes. Due to the changing age structure of the Bavarian population, 4.9 % of this population are estimated to live in nursing homes in 2025. These persons were not considered in the analyses. Based on demographic information and using age- and gender-stratified fracture rates, 17,119 femoral fractures are estimated to occur in the year 2014 and 21,263 femoral fractures in the year 2025. Of all femoral fractures in 2014 and 2025, 64.6 and 65.6 % of fractures were estimated to occur in persons with osteoporosis, respectively (Table 1*Estimate for the year 2014).*

In 2014, a moderate reduction of all femoral fractures by 10 % required 20.9 % of all community-dwelling persons aged 70–89 older to participate in FPE (Table 2). A substantial reduction by 25 % required a participation rate of 52.2 %. In order to lower the number of femoral fractures by 10 %, 36.9 % of persons with osteoporosis (N_osteoporosis_) needed to receive bisphosphonates. A reduction of all femoral fractures by 25 % required a treatment rate of 92.2 % (Table 2).

### Estimates for the year 2025

As displayed in Table 3, participation rates in FPE of 16.2 % would be necessary to limit the expected increase between 2014 and 2025 to 15 %. To keep the number of femoral fractures stable in 2025 compared to 2014, 42.7 % of all persons aged 70 to 89 years would need to participate in FPE in 2025. Estimates for the impact of bisphosphonate treatment demonstrated that 26.9 % of persons with osteoporosis would need to receive treatment in order to limit the expected increase of femoral fractures to 15 %. To keep the number of femoral fractures stable in 2025 compared to 2014, 70.8 % of persons with osteoporosis would need to receive bisphosphonate treatment.

### Sensitivity analyses

The calculations were repeated with the point estimate at the upper and lower boundary of the 95 % confidence intervals for RR (Tables 2 & 3). Using the lower boundary, 10 % reduction of femoral fractures in 2014 by FPE resulted in a required participation rate of 16.3 %, the upper boundary resulted in a required participation rate of 37.5 % and in required treatment rates of 26.7 and 77.4 %, respectively. To keep the number of femoral fractures stable between 2014 and 2025, using the lower boundary of the 95 % confidence interval required 33.4 % participation rate in FPE and 51.3 % treatment rate of osteoporosis; the upper boundary required 76.6 % participation in FPE, whereas even treatment of all persons with osteoporosis would not keep the number of femoral fractures stable.

## Discussion

In our model, between 2014 and 2025 the number of femoral fractures will increase by 24.2 % due simply to demographic changes - assuming that age- and sex-specific fracture rates are stable in this time period. Such projections have alerted policy makers in many countries and there is a growing need as well as interest in preventive measures to limit the upcoming burden for the health care systems. The objective of our model calculation was to estimate to theoretical impact of current available preventive measures on the burden of femoral fractures now and in the future.

Pillars of fracture prevention are fall-prevention strategies such as exercise programmes and the treatment of osteoporosis with anti-resorptive medications. About one third of all people aged 65 years and older have at least one fall each year and a past fall is a moderate predictor for future falls [23]. Therefore, falls are of relevance for most of the older people. Thus, fall prevention measures are justified in a broad range of the older population. Bisphosphonate treatment, however, is limited to patients with osteoporosis.

In our modelling approach, in order to achieve relevant changes in the absolute number of femoral fractures in 2014, a high participation rate in FPE of persons aged 70–89 years would be needed: 41.8 % of all persons in this age-group would need to participate in order to decrease the number of femoral fractures by 20 %. To lower the expected increase between 2014 and 2025, again high participation rates in FPE would be needed: an increase by 5 % in fractures instead of 24.2 % would require as many as 33.9 % of all community-dwelling older persons to participate in fall-prevention exercise. Translated to Bavaria’s largest city, Munich, to reduce the number of femoral fractures in Munich by 20 % in 2014 this translates to 80.000 persons training. Assuming that half of these persons would train at home and half in groups of 15 persons each, 2667 groups were required.

Although bisphosphonate treatment may be very effective only about 65 % of femoral fractures of persons 65 years and older are attributable to osteoporosis in our model. As a consequence, most persons with osteoporosis would need to receive bisphosphonates in order to reduce the number of femoral fractures: 79.4 % in 2014 and 56.2 % in 2025 to attain above mentioned reductions.

The calculations are based on meta-analyses pooling data from studies with various inclusion criteria. Effect size of FPE was taken from a meta-analysis that included participants with different fall risks at baseline. While some studies included participants only on the basis of age, other studies required the presence of specific risk factors [12]. However, fall risk at baseline does not seem to modify the effect size of FPE and was not considered in our analyses.

FPE studies conducted to date do not have sufficient power to use fractures as primary outcome variable. For our calculations a meta-analysis was used which demonstrated a reduction of all fractures combined, but did not report femoral fractures separately. However, femoral fractures represent the most frequent type of fragility fractures in older age and are nearly always the result of a fall. Therefore, it seems reasonable to assume a linear correlation between fracture incidence and femoral fracture incidence [1].

The effect size of bisphosphonate treatment on fracture reduction was taken from a meta-analysis including studies of four different oral bisphosphonates [13]. Inclusion criteria of these studies varied: some studies required a prior fracture, others recruited based on BMD with different T-score thresholds. In our analyses, we focused on persons aged 65 years and older with osteoporosis and did not consider persons with osteopenia. The incremental benefit of bisphosphonate treatment for persons with osteopenia is lower than for persons with osteoporosis. Including persons with osteopenia, the number of potentially preventable fractures would increase. At the same time, the proportion of persons that need to receive bisphosphonate treatment in order to prevent the targeted number of fractures would increase as well.

The estimated treatment and participation rates are far from the current situation. While some countries such as Australia and the UK may offer population-wide FPE classes, other countries such as Germany are only at the beginning of a wider implementation [24, 25]. Currently, FPE classes are sparse in Germany and there is no registry to estimate the number of classes or participants. The best acceptance of FPE has been observed in settings where FPE is an integral part of a population-based strategy to prevent falls [26]. However, even in countries with established structures such as Australia, a modelling calculation on the impact of population-wide implementation of Tai Chi found little impact on the absolute number of femoral fractures due to low uptake [27]. Uptake by older persons is hampered by numerous beliefs and attitudes [28]. Risk appraisal is particularly low in the “young old” while mobility problems often account for difficulties to reach the “oldest old” [29]. For these reasons we excluded these two age groups from our calculation of FPE participation.

Treatment of osteoporosis faces challenges as well. There is an on-going debate about the best screen-and-treat strategy [30, 31]. Still, osteoporosis is regarded as an under-diagnosed and under-treated condition and even amongst those at high risk of fractures, less than half receive specific medication [32–34].

Our modelling approach is optimistic in its conception for several reasons. First, we did not consider exclusion criteria for either FPE or bisphosphonate treatment. Furthermore, we did not assume any FPE or bisphosphonate treatment until beginning of the intervention in 2014. As for bisphosphonate treatment, it is estimated that about 10 % of persons with osteoporosis already receive bisphosphonate treatment in Germany [32]. In a sensitivity analysis we assumed identical age- and gender-specific treatment rates. As a result, the proportion of untreated persons with osteoporosis that need to receive bisphosphonate treatment would be slightly higher (e.g. 39.1 % instead of 36.9 % for a fracture reduction of 10 and 97.9 % instead of 92.2 % for a fracture reduction of 25 %). Since these assumptions are based on only vague data we did not include them in the final model. Structured FPE has not been implemented and reimbursed within the health care system until recently. There is no estimate on participation available due to the scarce availability of exercise classes and heterogeneity of providers. Local experts would estimate participation rates to be less than 1 %. As a consequence, we assumed that there was no relevant participation prior to 2014. In a sensitivity analysis we assumed again 10 % participation rates prior to 2014. Applying such an assumption, even more persons aged 70 to 89 years would need to practice FPE in 2014 in order to attain defined reductions in the number of femoral fractures (e.g. 29.6 % instead of 20.9 % for a fracture reduction of 10 % and 59.0 % instead of 52.2 % for a fracture reduction of 25 %).

Furthermore, we did not exclude those with contraindication to bisphosphonate treatment. Chronic kidney disease is the most relevant contraindication. Excluding persons with contra-indications for bisphosphonate treatment, again even higher treatment rates are needed in those eligible to achieve the defined reductions. Second, we assumed effect sizes as reported from trials. However, effect sizes in routine care may be lower than under study conditions due to a lower adherence or persistence to the prescribed treatment and interventions. As for bisphosphonate medication, there are analyses based on drug registries indicating poor persistence and adherence to bisphosphonate treatment that decrease effectiveness of bisphosphonate treatment [35, 36]. Whether new regimes such as once-yearly infusions will help to overcome the issue of adherence and persistence remains uncertain [37].

Furthermore, we assumed that age- and gender-specific fracture rates over time are influenced only by FPE or bisphosphonate treatment. Secular trends could decrease fracture rates in 2025 and could reduce the required participation and treatment rates. Interpreting the results with respect to FPE, one has to bear in mind that fall prevention offers benefits beyond fracture prevention like prevention of other fall-related injuries, reduction of fear of falling or social benefits.

The prevalence of osteoporosis was based on the Rotterdam study which provided age- and gender-specific rates of osteopenia and osteoporosis in a European population [20]. These rates, however, may not apply to populations from other regions of the world. Furthermore, the analyses included only community-living people. In Germany, about 20 % of all femoral fractures occur in institutionalised people [10]. To decrease the absolute number of femoral fractures across all populations, preventive efforts need to be extended to this relevant population.

## Conclusions

The burden of femoral fractures will increase considerably within the next years. Both bisphosphonates and FPE are effective measures to prevent femoral fractures. However, unrealistic high treatment and participation rates of these two interventions are needed to achieve substantial effects on the expected burden of femoral fractures in the total population of persons 65 years and older at present and in the future.

### Ethical approval

Not applicable.

### Consent for publication

Not applicable.

### Availability of data and materials

All data used in the calculations is found in the supplementary material, has been published, or is available to the public.

## Appendix 1

**Table 4:** Assumptions of the modelling

1	Age- and gender-specific rates of institutionalization are derived from 2009 and remain unchanged between 2009 and 2025. The rational for this assumption is the uncertainty of further development of age- and gender-specific institutionalisation rates and the limited impact of such changes on the absolute number of community-dwelling persons.
2	Age- and gender-specific fracture rates derived from routine data by AOK Bavaria applies to all other community-dwelling older persons living in Bavaria since more than 50 % of persons aged 65 and older are covered by this insurance.
3	Participation in FPE and bisphosphonate treatment between 2004 and 2009 were not considered, i.e. assumed to be 0 % since there is no valid data on true participation and treatment rates available.
4	Age- and gender-specific fracture rates derived from 01.01.2004 to 30.06.2009 remain unchanged until 2025.
5	Effect size of Fall Prevention Exercise (FPE) is based on a meta-analysis with ‘reduction of any fracture rate’ as endpoint. Effect size of the reduction of femoral fractures is identical since there is no other data available.
6	FPE targets only persons aged 70 to 89 years.
7	Effect size is constant over time for both interventions.
8	Age- and gender-specific distribution of osteoporosis remains unchanged until 2025.
9	Bisphosphonate treatment is limited to people with osteoporosis based on BMD values (T-score -2.5). Concomitant risk factors modifying the threshold for treatment are not considered.
10	The effect size of bisphosphonates in men is identical to that in women since data on effect size in men is rare.

## Appendix 2

**Table 5:** Age- and gender-specific rates of femoral fractures per 1000 person-years (community-dwelling persons only)

Age/gender	Men	Women
65–69	1.63	2.03
70–74	2.37	3.77
75–79	4.31	7.91
80–84	8.32	15.42
85–89	12.88	24.72
90+	24.59	39.99

Data derived and adapted from Rapp et al. [10]

## Appendix 3

**Table 6:** Age- and gender-specific distribution of osteoporosis and osteopenia

	Normal BMD		Osteopenia		Osteoporosis	
	Men	Women	Men	Women	Men	Women
Age	[%]	[%]	[%]	[%]	[%]	[%]
65–69	20.1	22.3	65.8	60.5	14.1	17.2
70–74	22.3	15.5	64.4	62.2	13.3	22.3
75–79	20.9	12.2	59.9	57.8	19.2	30.0
80–84	19.2	11.3	58.3	54.4	22.5	34.3
85+	12.1	6.5	51.5	48.4	36.4	45.2

Data from Rotterdam study, details by personal communication [20]

## Abbreviations

FPE: fall prevention exercise
BMD: bone mineral density
N_all_: number of community-dwelling persons
FF_all_: total number of femoral fractures
FR_all_: rates of femoral fractures
PFF: number of femoral fractures to be prevented
N_osteoporosis_: number of persons with osteoporosis
FF_osteoporosis_: number of femoral fractures in persons with osteoporosis
FR_osteoporosis_: rates of femoral fractures in persons with osteoporosis
N_70–89_: number of persons aged 70 to 89 years
FF_70–89_: femoral fractures in persons aged 70 to 89 years
FR_70–89_: rates of femoral fractures in persons aged 70 to 89 years
